# Accurate position exchange of stamen and stigma by movement in opposite direction resolves the herkogamy dilemma in a protandrous plant, *Ajuga decumbens* (Labiatae)

**DOI:** 10.1093/aobpla/plz052

**Published:** 2019-08-17

**Authors:** Zhong-Ming Ye, Xiao-Fang Jin, Jian Yang, Qing-Feng Wang, Chun-Feng Yang

**Affiliations:** 1 CAS Key Laboratory of Aquatic Botany and Watershed Ecology, Wuhan Botanical Garden, Chinese Academy of Sciences, Wuhan, China; 2 Institute of Ecology and Environmental Science, Nanchang Institute of Technology, Nanchang, China

**Keywords:** *Ajuga*, dichogamy, evolution, generalist pollination, herkogamy, pollination accuracy, protandry, sexual interference

## Abstract

Herkogamy is an effective way to reduce sexual interference. However, the separation of stigma and anther potentially leads to a conflict because the pollen may be placed in a location on the pollinator different from the point of stigma contact, which can reduce pollination accuracy. Floral mechanisms aiming to resolve this conflict have seldom been explored. The floral biology of protandrous *Ajuga decumbens* was studied to uncover how the herkogamy dilemma can be resolved. Flower anthesis was divided into male, middle, female and wilting phases. The positions of stigma and stamen were dissimilar in different flower development stages. We measured the distance of the stamen and stigma to the lower corolla lip at different floral phases, which was the pollinators’ approaching way. The pollen viability, stigma receptivity, pollen removal and pollen deposition on stigma were investigated at different phases. During the male phase, the dehisced anthers were lower than the stigma, located at the pollinators’ approaching way, and dispersed most pollen with high viability. As the flower developed, the anthers moved upwards, making way for pollen deposition during the female phase. Meanwhile, the stigma becomes receptive by moving into the way and consequently was deposited with sufficient pollen. The position exchange of the stamen and stigma created a dynamic herkogamy at the floral phase with different sexual functions. This floral mechanism effectively avoided sexual interference and maintained pollination accuracy. In *Ajuga*, the movement herkogamy might be of adaptive significance in response to the changes in the pollination environment.

## Teaser

Herkogamy may successfully avoid sexual interference but may also lead to reduction of pollination accuracy. The floral mechanism of protandrous *Ajuga decumbens* has perfectly resolved the herkogamy dilemma by putting the stamen and stigma in the same place but at times of different sexual functions. The movement of the stamen and stigma in opposite directions results in position exchange in the male and female floral phases. The interactions of dynamic herkogamy and dichogamy not only completely avoid sexual interferences, but also ideally maintain pollination accuracy. This novel floral mechanism highly enriches our understanding on the evolution of herkogamy.

## Introduction

Herkogamy and dichogamy are mechanisms of hermaphroditic angiosperms to spatially and temporally separate pollen presentation and stigma receptivity. The two mechanisms may collectively or separately work to promote outcross mating and/or reduce sexual interference (Lloyd and Webb 1986; Webb and Lloyd 1986; Barrett 2002a; Li *et al.* 2013). A dichogamous flower can effectively avoid self-pollination and enhance outcross mating due to the separation of the male and female function within the flower (Lloyd and Webb 1986; Bertin and Newman 1993; Barrett 2002a). For homostylous flowers without assortative mating mechanism (such as, heterostylous flower), herkogamy is mainly thought to reduce interference between male and female functions (Lloyd and Webb 1986; Webb and Lloyd 1986; Barrett 2002a, b; Li *et al.* 2013). Nevertheless, excessive herkogamy may greatly reduce the possibility of the contact of pollinators with both the sexual organs, while scanty herkogamy may induce physical interference as male and female organs to block each other’s access to pollinators (Webb and Lloyd 1986; Barrett 2002a).

Lloyd and Webb (1986) recognized that herkogamy might effectively avoid sexual interference but it might also result in a conflict due to the spatial separation of male and female functions, which can minimize the pollination accuracy in hermaphroditic plants. Pollen may be placed in a site on the pollinator different from the point of stigma contact. Armbruster *et al.* (2009) pointed out that dichogamy and herkogamy with higher dimensional space were two possible routes to escape from the trade-off between pollination accuracy and classical herkogamy (e.g. approach herkogamy and reverse herkogamy). Floral mechanisms through interactions of herkogamy and dichogamy may decrease the interference between pollen removal and pollen receipt, thus maintaining the pollination accuracy (Medan and Ponessa 2003; Armbruster *et al.* 2014; Ganie *et al.* 2015).

An ideal floral mechanism to resolve herkogamy dilemma is that male and female functions separate in time and moreover, stamen and stigma sequentially occupy the same position for pollination at male and female phases. For example, the stamen locates a spot with high pollination accuracy during the male phase, while the stigma remains away from the position to make way for pollen export. During the female phase, the stigma and stamen exchange their positions in order to place the stigma in the accurate point for pollen deposition. This mechanism of movement herkogamy could be achieved by successive stamen movement (Ren and Tang 2012; Armbruster *et al.* 2014; Xiao *et al.* 2017) or by elongation of the style (Guo *et al.* 2014; Mamut *et al.* 2014). In this study, we reported a floral mechanism of movement herkogamy in a protandrous herb, *Ajuga decumbens*, where the stamen and stigma of a flower exchanged their position by movement in opposite directions with floral development. To detect whether and how this floral mechanism avoided sexual interference, and maintained the pollination accuracy, the reproductive ecology of *A. decumbens*, the dynamic performance of male and female functions and the effects of this floral mechanism on pollen removal and deposition were studied.

## Materials and Methods

### Study species and site

**Figure 1.**
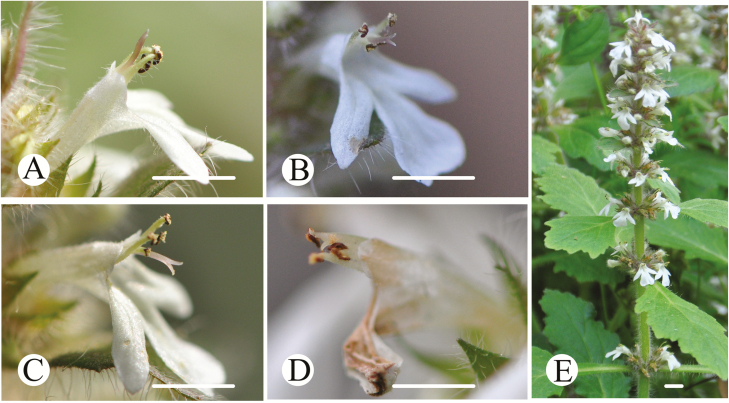
Floral morphology and inflorescence trait of *Ajuga decumbens*. (A) to (D) indicate male phase, middle phase, female phase and witling phase, respectively, while (E) shows the inflorescence trait. Scale bar = 1 cm.

*Ajuga decumbens* is an annual or biennial herb endemic to China. The plant is ~10–30 cm in height and is widely distributed in the south area of the Yangtze River. It inhabits streamsides, roadsides and wet areas. Individual plants produce multiple verticillasters (12.17 ± 0.42, *n* = 60) that arise from the grass roots, and each inflorescence has 5–12 crowded whorls bearing 2–16 white flowers (Fig. 1). Flowers are zygomorphic and nectar-rich. The calyx consists of five nearly equal lobes with densely pilose, while the corolla tube is nearly twice as long as the calyx with sparsely pilose. The corolla has a very short two-lobed upper lip and a three-lobed lower lip (Fig. 1). Each flower produces four epipetalous stamens arranged in two rows, four ovules and a bifid stigma (Wu and Li 1977; Fig. 1). The anthers dehisce longitudinally. The flowering season of this species in China is usually from March to June.

All experiments were conducted in a natural population of *A. decumbens* at Wuhan Botanical Garden (30.551°N, 114.429°E; 23 m a.s.l.), Hebei province, China. Before flower anthesis, 100 plants from the natural population were randomly selected and transplanted into pots on 15 February 2013. Each individual was planted in a 25-cm pot filled with the original surface soil from the natural population area. All the plants were managed under the same conditions, for example, with the same soil and water source. They were initially bagged with nylon net mesh to keep them away from any kind of insects by the side of the natural population. The transplanted plants were watered once every 3 days during our investigations. The transplanted plants were selected for further investigations if they showed similar performance as those of natural population in plant height, number of inflorescences per plant and number of flowers per inflorescence.

### Pollination and mating system

To explore the pollinator composition of *A. decumbens*, six transplanted plants were used to set a plot near the natural population for observation. On sunny days, i.e. from 27 to 29 March in 2013, we renewed all the plants of the plot every day and conducted observations from 09:00 am to 4:30 pm. Pollination observation was carried out during a period of 10 min. A total of 24 observation periods were conducted (eight periods on each observation day). The pollinators whose bodies contacted the reproductive organs (stigma or anthers) were recorded and the number of flowers visited by each pollinator in the plot was also noted. We traced the pollinator that visited a plot to record the number of flowers visited on each plant and each inflorescence. For each pollinator, the visited flowers per plant and inflorescence within a single foraging bout were calculated. The pollinators were collected and identified by the Institute of Zoology, Chinese Academy of Sciences.

To reveal the mating system of *A. decumbens*, three pollination treatments were conducted on the bagged individuals: (i) emasculation and hand-pollination with pollen grains from other plants; (ii) emasculation and hand-pollination with pollen from the same plant; and (iii) the bagged flowers without emasculation and pollination. Ten inflorescences for each treatment were selected from 10 plants (30 inflorescences were selected in total); five flowers per inflorescence were used for hand-pollination with self- or cross-pollen in treatment (i) and (ii), all flowers of the inflorescence were used to test autonomous selfing in treatment (iii). To ensure sufficient pollination, all the flowers were hand-pollinated twice each day on two consecutive days during the female phase. In addition, 10 plants were placed into the natural population for open pollination and this served as a control (one inflorescence per plant was selected for calculating fruit set and seed set). The average total number of flowers per inflorescence in the treatments for testing autonomous selfing and open pollination were 47.25 ± 2.60. Fruit set was calculated as the fruit number divided by the number of flowers. A generalized linear model with binomial error structure was used to assess the effects of pollination treatment on the fruit set. A Poisson generalized linear model was used to test the effects of pollination treatment on the seed number per fruit in a fixed model.

### Floral anthesis and pattern of dichogamy and herkogamy

To detect the dynamic changes of *A. decumbens* flower during anthesis under bagged and natural conditions, 10 buds from five individuals were labelled as both bagged and natural plants. The flower status was monitored twice every day from open to wilt, especially on the position of stigma and stamens, the anther dehiscence and the status of stigma lobes. According to the relative position of the stigma and stamens, the floral development was divided into four phases: (i) male phase, indicating that the anthers are dehiscing meanwhile the stigma is higher than the stamens, the stigma lobes are close; (ii) middle phase, indicating that the stigma and stamens are at the same height and the stigma lobes are ready to spread; (iii) female phase, indicating that the stigma lobes are fully opened and lower than the anthers; and (iv) wilting phase, indicated by the wilted corolla and withered anthers and stigma. During this process, the stamen and stigma move in the opposite direction and exchange their positions. To precisely evaluate the position of the stamen and stigma, the distance from the stamen and the stigma to the lower lip was measured at the male, middle and female phases, respectively. We set the lowest point of the lowest anther and the lower lobe of stigma as the starting point, respectively. The symmetry line on the lower lip surface was set as the ending line and the shortest distance from the starting point to this line on the lower lip surface was measured by an electronic Vernier caliper (mm; see also Fig. 1). Thirty flowers at each phase were picked for measurement of such distance, respectively. Independent *T*-test was used to detect the differences in the distance from the stamen and the stigma to the lower lip among the three phases.

### Pollen viability and stigma receptivity

To test the changes in male and female functions and their relation to different floral phases, we tested stigma receptivity and pollen viability using the flowers from bagged individuals. In each floral phase, the stigmas of 15 flowers from different plants were collected to test the receptivity. The stigmas were stained by MTT (3-(4,5-dimethylthiazol-2-yl)-2,5-diphenyltetrazolium bromide). Afterwards, the dark or brown spots on the stigma revealed the presence of dehydrogenase, which in turn was used as an indicator for stigma receptivity (Dafni 1992). The ratio of stigma receptivity was calculated as the number of stained stigmas divided by the number of the total checked stigmas. In each floral phase, the pollen grains of six flowers from different plants were collected to evaluate the pollen viability. The pollen grains were treated with 10 % sucrose and 0.03 % boric acid solution for 2 h at 25 °C (Dafni 1992). The pollen germination ratio was then checked by an epifluorescence microscope (Nikon E-600). The ratio of the pollen grains with pollen tubes out of the total pollen grains showed the pollen viability. One-way ANOVA was conducted to detect the differences in the ratio of pollen viability among the four floral phases. To satisfy the normal distribution, pollen germination ratio was transformed by arcsine. Student–Newman–Keuls was used for multiple comparisons.

### Pollen removal at different floral phases

To investigate the dynamic pollen removal across the floral phases, we examined the remaining pollen before and after the style defluxion under open pollination. A total of 120 buds were randomly selected from 15 plants (eight from each plant) and divided into four groups (30 flowers for each group) according to the floral development phase, namely, bud phase, male phase, middle phase and female phase. The anthers of the labelled flowers from each group were collected at the end of the corresponding floral phase. The 15 plants were placed into the natural population during the experimental period. All the collected anthers from each flower were stored in 1 mL of 70 % ethanol and loosened by sonication. To estimate the remaining pollen in each flower, the pollen count of five subsamples of 20 μL each were averaged and multiplied by 50 (the total volume). One-way ANOVA was conducted to detect the differences in the remaining pollen among the four phases, and Student–Newman–Keuls was used for multiple comparisons.

### Pollen deposition at different floral phases

To investigate the dynamic pollen deposition across the floral phases, we compared the pollen deposition on the stigma of emasculated flowers and intact flowers under open pollination. Emasculation was done by clipping off all the anthers prior to the opening of the flowers, and intact flowers were set as control lines. A total of 240 flowers from 30 plants (eight from each plant) were arranged into two sections: 120 were emasculated and another 120 were not emasculated. The emasculated and intact flowers were divided into four groups (30 in each group) according to the four floral phases. The 30 plants were placed into the natural population during the experimental period. The stigmas were collected at 08:00 am in the next morning at the end of each floral phase. They were fixed in FAA solution (formalin:acetic acid:70 % ethanol at a ratio of 5:5:90 by volume). Stigmatic pollen load was counted under a fluorescence microscope (Nikon E-600) after treating them with 8 mol L^−1^ NaOH for 10 h, followed by 0.1 % aniline blue dye. A generalized linear model was used to test the effects of floral phase and emasculated treatment on pollen deposition in a fixed model under normal distribution. Floral phase and emasculated treatment were set as fixed factors.

All the data in this study were analysed using SPSS 22 statistical software (IBM Corp., Armonk, NY, USA). All values were presented as mean ± standard error.

## Results

### Pollination and mating system

In total, 101 individual insects from 10 species belonging to two orders and four families visited *A. decumbens.* Actually, only one species belonged to Lepidoptera and the other nine species belonged to Apoidea. *Osmia rufina*, *Tetralonia chinensis*, *Habropoda bucconis* and *Apis mellifera* were the frequent pollinators. All the pollinators were larger than a single *A. decumbens* flower in size **[see****Supporting Information—Fig. S1****]**. They headed into the corolla tube and probed the floral base for nectar. As the pollinator sought for nectar, the front part of its head rubbed against the anthers at the male phase and was deposited with pollen grains while at the female phase, stigma lobes contacted the similar part of the pollinator’s body and picked up the pollen grains **[see****Supporting Information—Fig. S1****]**. The average visited flowers per plant and inflorescence within a single foraging bout were 3.85 ± 0.33 (*N* = 171) and 1.83 ± 0.068 (*N* = 359), respectively.

There was no significant difference in the fruit set among hand-pollination with outcross- (98 %) and self- (90 %) pollen grains and under open pollination (95.14 %) (χ ^2^ = 3.19, *P* = 0.20). The bagged flowers without pollination set very few fruits (3.94 %). Seed set did not differ among hand-pollination with outcross- (86.22 %) and self- (90.56 %) pollen grains and under open pollination (95 %) (χ ^2^ = 2.00, *P* = 0.37) [see Supporting Information—Table S1].

### Flower anthesis and patterns of dichogamy and herkogamy

**Figure 2.**
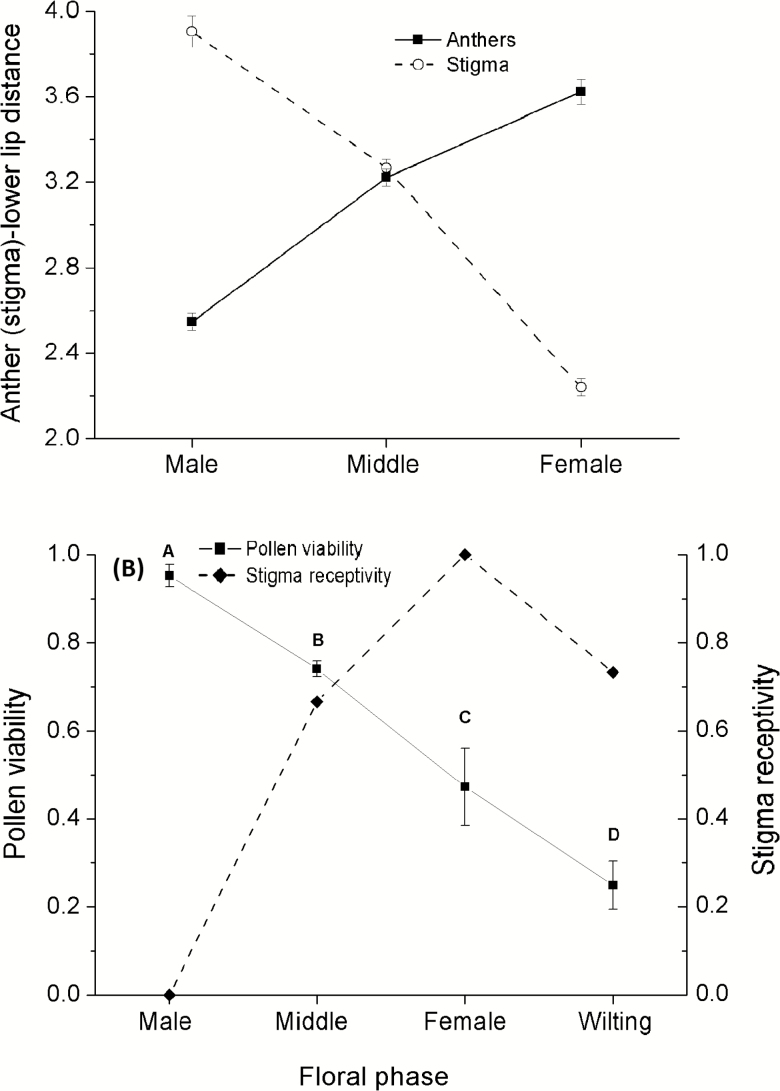
The position of stamen and stigma and pollen viability and stigma receptivity at different floral phases in *Ajuga decumbens*. (A) indicating the height of stamen (solid line) and stigma (dotted line) to the lower lip of the corolla at different floral phases and (B) indicating pollen viability (solid line) and stigma receptivity (dotted line). Different letters indicate significant differences at *P* < 0.05.

The flower longevity of *A. decumbens* was 4 days under natural conditions and each floral phase under natural conditions lasted for ~24 h. However, a bagged flower lasted for 8 days, with each floral phase of ~48 h. Particularly, the stigma maintained high receptivity (73.33 %) during the wilting phase under bagged conditions, while it wilted under natural conditions. Incomplete dichogamy and movement herkogamy were found in *A. decumbens*. The dichogamy of *A. decumbens* was protandry, where the male function preceded the female function. The movement herkogamy of *A. decumbens* was performed as the stigma deflexed to a lower location after opening and the stamens flexed up to a higher location after the anthers’ dehiscence. During the male phase, the stigma was higher than the stamens (*T* = 17.17, *P* < 0.001; 3.91 ± 0.07 mm vs. 2.55 ± 0.03 mm), although the stigma lobes were not opened, and the anthers were dehiscing (Fig. 1A). During the middle phase, the stigma and the stamen were close to each other and were almost at the same height (*T* = 1.47, *P* = 0.15; 3.27 ± 0.02 mm vs. 3.22 ± 0.02 mm), while the stigma lobes were opening, and the anthers were dehiscing (Fig. 1B). During the female phase, the stigma was lower than the stamen (*T* = −21.69, *P* < 0.001; 2.24 ± 0.02 mm vs. 3.62 ± 0.06 mm), whereas the stigma lobes were fully opened, and the anthers were almost without pollen (Fig. 1C). From the male to the female phase, the stamen and stigma completely exchanged their positions (Fig. 2A). During the wilting phase, the stigma and the stamen maintained their positions as in the female phase, while the floral organ was wilting (Fig. 1D) [see Supporting Information—Table S1].

### Pollen viability and stigma receptivity

Pollen viability was significantly different among the four floral phases (*F*_3, 21_ = 32.75, *P* < 0.001), and the pollen viability was near 100 % during the male phase and was decreased notably with the floral development phases (Fig. 2B). However, MMT staining indicated that the stigma had no receptivity during the male phase but displayed receptivity during the middle phase (66.67 %) and was near 100 % during the female phase (Fig. 2B) [see Supporting Information—Table S1].

### Pollen removal and deposition at different floral phases

**Figure 3.**
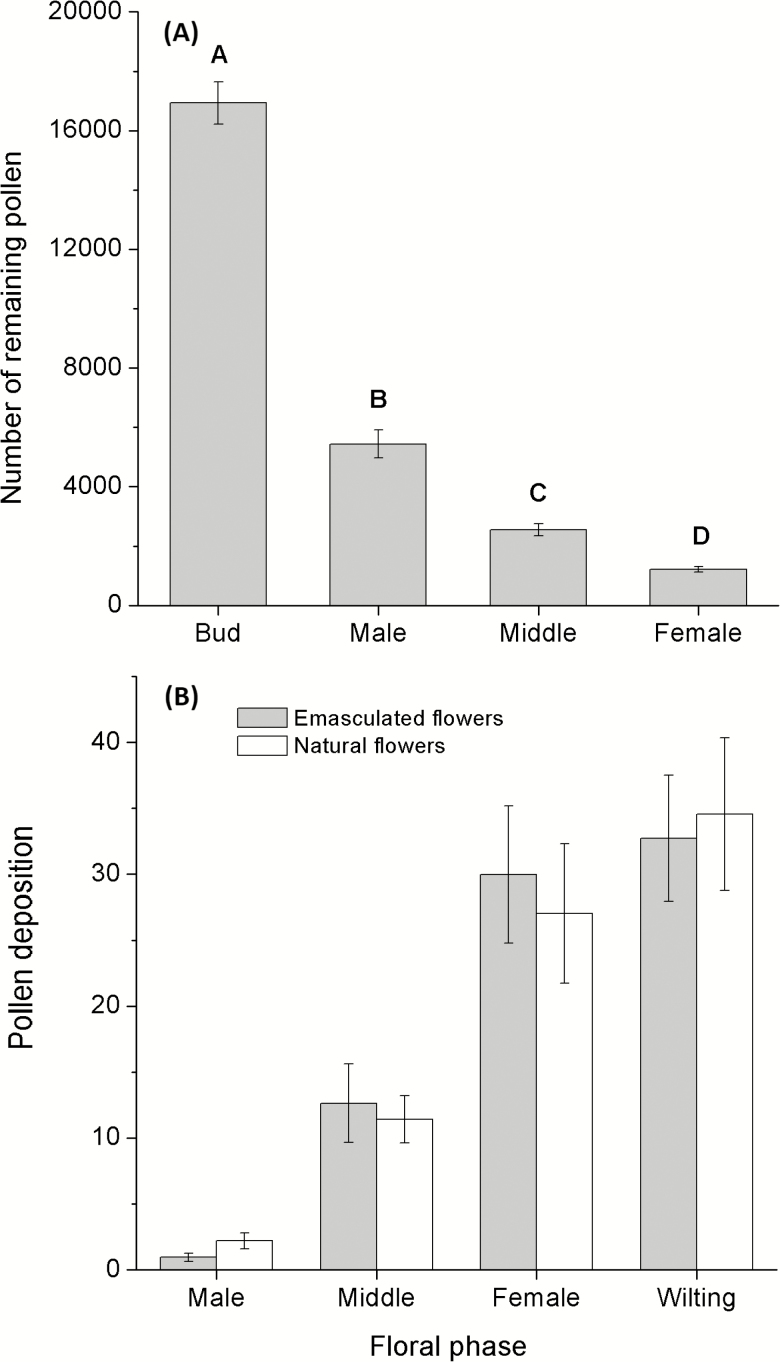
Pollen removal and deposition at different floral phases in *Ajuga decumbens*. (A) indicating pollen remains and (B) showing stigmatic pollen loads for emasculated flowers (grey column) and intact flowers (white column) under open pollination. Different letters indicate significant differences at *P* < 0.05.

When a pollinator visited a flower at the male phase, dehiscing anthers released pollen grains on the front part of its head; meanwhile, the non-receptive stigma located outside of the point of pollinator–anther contact. At the female phase, the dehisced anthers and the receptive stigma exchanged position; the stigma contacted with the front part of the head of a visiting pollinator and was deposited with pollen grains while the dehisced anthers located outside of the interaction. The amount of remaining pollen during the four phases was significantly different (*F*_3, 116_ = 258.55, *P* < 0.001; Fig. 3A). The total number of pollen grains of *A. decumbens* was 16 933 ± 716.54. More than 80 % of the pollen grains were removed at the end of the male phase (Fig. 3A). On the pollen deposition, the emasculated flowers showed no significant difference when compared with intact flowers (χ ^2^ = 0.01, *P* = 0.92). However, the flowers during the male phase received very few pollen grains (Fig. 3B). The pollen deposition on the stigmas of the flowers during the four floral phases was significantly different (χ ^2^ = 87.32, *P* < 0.001; Fig. 3B). The stigmatic pollen load was increased with the sequence of the floral developing phases [see Supporting Information—Table S1].

## Discussion

The floral mechanism of protandrous *A. decumbens* perfectly resolved the herkogamy dilemma by putting the stamen and stigma in the same place but at times of different sexual functions. The movement of the stamen and stigma in opposite directions resulted in the exchange of position at the male and female floral phases. The interactions of dynamic herkogamy and dichogamy not only completely avoided sexual interference but also ideally maintained pollination accuracy. This floral mechanism well resolved the herkogamy dilemma, which enriched our understanding of the evolution of herkogamy (see also Armbruster *et al.* 2014).

The position exchange of the stamen and stigma of *A. decumbens* was achieved by style bending downwards and filament bending upward, causing opposite movement of anthers and stigma. A dynamic movement of style has been found to play some roles in plant reproduction, e.g. enhancing of the pollination in Columbine (Yu and Huang 2006), and the formation of an assortative mating system in gingers (Li *et al.* 2001). The stamen movement due to the sequential maturation of anthers was also thought to be beneficial for the reproductive success of the plant (Aizen and Basilio 1995; Medan and Ponessa 2003; Armbruster *et al.* 2014; Xiao *et al.* 2017). In *A. decumbens*, all the anthers matured simultaneously. With the flower development, the filament was bending upwards with the anther dehiscing, and the style was bending downwards with the stigma becoming receptive. Compared to the male phase, the stamen and stigma completely exchanged their positions at the female phase and created a dynamic herkogamy at the floral phase with different sexual functions. The anthers at the male phase and stigma at the female phase were in contact with the same location on the front part of the pollinator’s head. The floral mechanism thus made the dehiscing anthers and receptive stigma to occur in the right place and at the right time (Armbruster *et al.* 2014).

During the male phase, *A. decumbens* flower made way for pollen removal when the anthers were ready for releasing pollen with high viability. Pollen removal might be significantly reduced by the stigma when it became a physical obstruction of the pollinators’ approaching way (Schlessman 1986; Kohn and Barrett 1992; Fetscher 2001; Parra-Tabla and Bullock 2005). However, the stigma of *A. decumbens* flower was out of the way for pollinators’ approaching, avoiding any interference with pollen removal during the male phase. This might contribute to the high efficiency of pollen removal, where >80 % of pollen was removed at this stage. At the end of the male phase, the dehisced anthers moved upwards, leaving the pollinators’ approaching way, while the stigma moved downwards. They completely exchanged their locations during the female phase when the stigma became receptive and ready for receiving pollen grains. Without the obstruction of the stamen, a large amount of pollen grains was deposited on the receptive stigma during the female phase. It was, therefore, suggested that the floral mechanism well contributes to pollen removal at the male phase and deposition on stigma at the female phase.

A previous study of another species of the genus (*A. bracteosa*), which occurred in pollinator-rare conditions, showed that style elongation together with bending of one of the stigma branches assisted self-pollination (Ganie *et al.* 2015). Although self-pollination might occur during the middle phase in *A. decumbens*, the spatial separation in the anther and the stigma might restrict the occurrence of self-pollination as emasculation did not reduce pollen deposition during most of the phases (see also Verma *et al.* 2004). In our study site, the plants attracted many pollinators. Moreover, during the female phase, there was an increase in the nectar replenishment, which may maintain the flowers’ attraction to pollinators as most of the pollen grains were removed (Z.-M. Ye, unpubl. data). In *Ajuga*, the floral mechanism of dynamic herkogamy by position exchange of the stigma and the stamen might be of adaptive significance in response to the changes in the pollination environment.

## Supporting Information

The following additional information is available in the online version of this article—

**Figure S1.** Pollinators visiting *Ajuga decumbens* flowers. (A) *Tetralonia chinensis*; (B) *Habropoda bucconis*; (C) *Apis mellifera*; (D) *Osmia rufina*. All the pollinators are larger than a single *A. decumbens* flower in size; the contact area to anthers and stigma is the front head for all the insects. Scale bar = 1 cm.

**Table S1.** The original data for this article.

plz052_suppl_Supplementary_Figure_Legend

plz052_suppl_Supplementary_Figure_S1

plz052_suppl_Supplementary_Table_S1

## Sources of Funding

The research was supported by the National Natural Science Foundation of China to C.-F.Y. (grant no. 31770255).

## Contributions by the Authors

Z.-M.Y. and C.-F.Y. designed the research project; Z.-M.Y., J.Y. and X.-F.J. performed the experiments; Z.-M.Y., X.-F.J. and C.-F.Y. analysed the data; Z.-M.Y. and C.-F.Y. wrote the paper. All authors contributed to the final manuscript.

## Conflict of Interest

None declared.
