# Supplementary material for: Accurate position exchange of stamen and stigma by movement in opposite direction resolves the herkogamy dilemma in a protandrous plant, Ajuga decumbens (Labiatae)
Source: AoB Plants. 2019 Aug 17;11(5):plz052. doi: 10.1093/aobpla/plz052 (PMC6757348; doi:10.1093/aobpla/plz052)
Supplement: plz052_suppl_Supplementary_Figure_Legend [file plz052_suppl_supplementary_figure_legend.docx]

**Supplemental Figure legend**

**Fig. S1**. Pollinators visiting *Ajuga decumbens* flowers. A. *Tetralonia chinensis*; B. *Habropoda bucconis*; C. *Apis mellifera*; D. *Osmia rufina*. All the pollinators are larger than a single *A. decumbens* flower in size; the contact area to anthers and stigma is the front head for all the insects. Scale bar = 1 cm.
