# Supplementary figures and images for: Accurate position exchange of stamen and stigma by movement in opposite direction resolves the herkogamy dilemma in a protandrous plant, Ajuga decumbens (Labiatae)
Source: AoB Plants. 2019 Aug 17;11(5):plz052. doi: 10.1093/aobpla/plz052 (PMC6757348; doi:10.1093/aobpla/plz052)

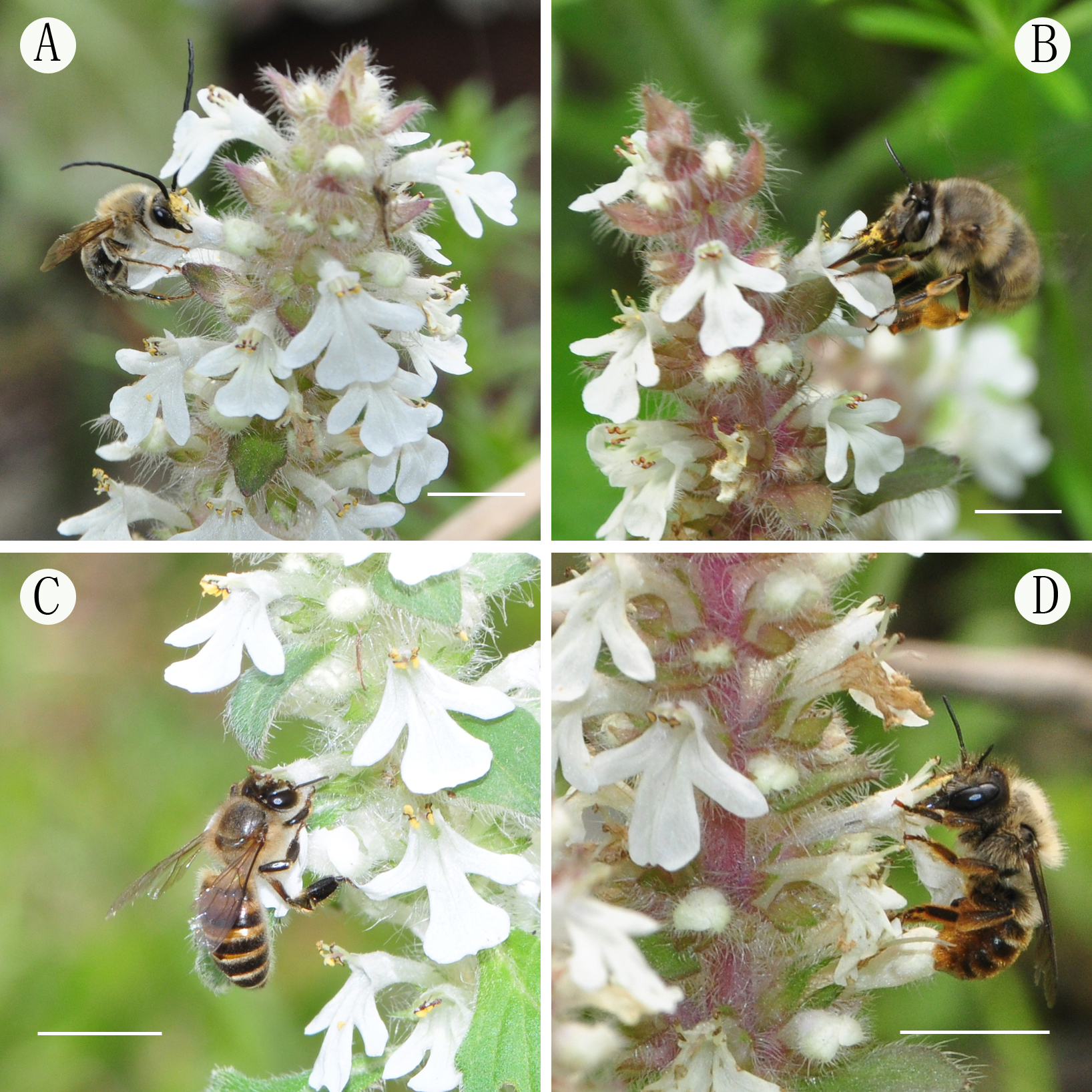

Supplement: plz052_suppl_Supplementary_Figure_S1 [file plz052_suppl_supplementary_figure_s1.jpeg]
